# Distinct impacts of fat and fructose on the liver, muscle, and adipose tissue metabolome: An integrated view

**DOI:** 10.3389/fendo.2022.898471

**Published:** 2022-08-17

**Authors:** Maria João Meneses, Inês Sousa-Lima, Ivana Jarak, João F. Raposo, Marco G. Alves, Maria Paula Macedo

**Affiliations:** ^1^ iNOVA4Health, NOVA Medical School/Faculdade de Ciências Médicas (NMS/FCM), Universidade Nova de Lisboa, Lisbon, Portugal; ^2^ Portuguese Diabetes Association - Education and Research Center (APDP-ERC), Lisbon, Portugal; ^3^ Department of Pharmaceutical Technology, Faculty of Pharmacy, University of Coimbra, Coimbra, Portugal; ^4^ Department of Anatomy and Unit for Multidisciplinary Research in Biomedicine (UMIB), Institute of Biomedical Sciences Abel Salazar (ICBAS), University of Porto, Porto, Portugal; ^5^ Medical Sciences Department, University of Aveiro, Aveiro, Portugal

**Keywords:** prediabetes, non-alcoholic fatty liver disease, diet, metabolomics, muscle, adipose tissue

## Abstract

**Objective:**

In the last years, changes in dietary habits have contributed to the increasing prevalence of metabolic disorders, such as non-alcoholic fatty liver disease (NAFLD) and type 2 diabetes mellitus (T2DM). The differential burden of lipids and fructose on distinct organs needs to be unveiled. Herein, we hypothesized that high-fat and high-fructose diets differentially affect the metabolome of insulin-sensitive organs such as the liver, muscle, and different adipose tissue depots.

**Methods:**

We have studied the impact of 12 weeks of a control (11.50% calories from fat, 26.93% from protein, and 61.57% from carbohydrates), high-fat/sucrose (HFat), or high-fructose (HFruct) feeding on C57Bl/6J male mice. Besides glucose homeostasis, we analyzed the hepatic levels of glucose and lipid-metabolism-related genes and the metabolome of the liver, the muscle, and white (WAT) and brown adipose tissue (BAT) depots.

**Results:**

HFat diet led to a more profound impact on hepatic glucose and lipid metabolism than HFruct, with mice presenting glucose intolerance, increased saturated fatty acids, and no glycogen pool, yet both HFat and HFruct presented hepatic insulin resistance. HFat diet promoted a decrease in glucose and lactate pools in the muscle and an increase in glutamate levels. While HFat had alterations in BAT metabolites that indicate increased thermogenesis, HFruct led to an increase in betaine, a protective metabolite against fructose-induced inflammation.

**Conclusions:**

Our data illustrate that HFat and HFruct have a negative but distinct impact on the metabolome of the liver, muscle, WAT, and BAT.

## Introduction

Dysmetabolism drives obesity and/or diabetes as a result of excessive and abnormal accumulation of body fat impinging on several adverse health effects (1). Although awareness for these health and social problems has increased over the years, its prevalence continues to increase dramatically (2). Genetic background influences dysmetabolism development (3), yet the vast majority of the cases are due to factors that arose in industrialized countries, namely, excessive consumption of highly processed and energy-dense foods, frequently combined with a sedentary lifestyle. Indeed, these diets are usually rich in fructose and fat. While fructose is known to promote hepatic *de novo* lipogenesis, lipid accumulation, and insulin resistance (4), high-fat diets lead not only to hepatic lipid accumulation but also to higher adipose tissue lipolysis (5). However, fats and specifically triglycerides are differently metabolized depending on the fatty acid chain length. While medium-chain triglycerides are absorbed into the portal circulation and transported to the liver, long-chain fatty acids are transported through the lymphatic system *via* chylomicrons (6).

The metabolic and homeostatic dysfunctions (7) caused by the poor lifestyle habits may end up in the onset of hypertension, type 2 diabetes mellitus (T2DM) (8), cardiovascular disease, dyslipidemia (9), and non-alcoholic fatty liver disease (NAFLD) (10). The latter is characterized by the presence of macrovesicular steatosis in ≥5% of hepatocytes, in individuals with no causes for secondary hepatic accumulation, as is the case of abusive alcohol consumption and hereditary disorders (11). NAFLD is the most common cause of chronic liver disease in Western countries, having a reported prevalence of 6%–35% worldwide (12). Although the molecular mechanisms leading to NAFLD are still under discussion, it is known that it is a consequence of several factors, namely, (i) an increased incursion of free fatty acids from insulin-resistant adipose tissue, (ii) impaired metabolism of dietary lipids in the liver or impaired lipid export from the hepatocytes, and (iii) increased *de novo* lipogenesis in the liver (13). The overload of lipids affects not only the liver but also other metabolic organs such as the muscle or adipose tissue (both white and brown). Considering organ crosstalk, dysfunctions in one of these organs will have an impact on others, namely, in cases of insulin resistance and inflammation, and dysmetabolism overall (14, 15).

We hypothesized that dietary content in lipids or fructose differentially affect the metabolome of insulin-sensitive organs. Our aim was to evaluate the metabolome of insulin-sensitive organs, as is the case of the liver, muscle, and white and brown adipose tissue in animals fed with high-fat or high-fructose diet.

## Materials and methods

### Chemicals

Bicinchoninic acid (BCA) Protein Assay Kit was purchased from Thermo Scientific (Waltham, MA, USA). Dried milk was purchased from Nestlé (Vevey, Switzerland). Amersham ECL was purchased from GE Healthcare (Weßling, Germany). NZYColour Protein Marker II, agarose, NZYDNA Ladder V, and Supreme NZYTaq II 2x Green Master Mix were purchased from NZYTech (Lisbon, Portugal). Fructose was purchased from Enzymatic (Loures, Portugal). Immobilon-P polyvinylidene difluoride (PVDF) membrane was purchased from Merck Millipore (MA, USA). Kits for triglycerides and cholesterol determination were purchased from Spinreact (Girona, Spain). Quantification of free fatty acids was done using a kit from Wako Diagnostics (CA, USA). All other chemicals were purchased from Sigma-Aldrich (St. Louis, MO, USA), unless stated otherwise.

### Animals

Experiments were performed using male C57Bl6/J mice kept on a 12-h light/dark cycle with *ad libitum* access to food and water. Animals were randomly divided in three groups (n=7/group) and fed different diets from 6 to 18 weeks of age: normal chow diet with 11.50% calories from fat, 26.93% from protein, and 61.57% from carbohydrates [Chow group; RM3A(P), Special Diets Services, Witham, Essex, UK]; high-fat diet with 58% of calories from fat, 16.4% from protein, and 25.5% from carbohydrates (HFat; D12331, Research Diets, New Brunswick, NJ) or high fructose diet [HFruct; FRUC-00T-500, Enzymatic, PT; 35% w/v in drinking water and fed with RM3A(P) diet]. Mice were monitored weekly for body weight, blood glucose levels, and for distress signals. The experimental procedures were approved by the Ethics Committee of the NOVA Medical School and by the Directorate-General for Food and Veterinary that regulates the animal care and use in research (registration number 82/2019/CEFCM and 0421/000/000/2016, respectively). All procedures followed Animal Research: Reporting of *In Vivo* Experiments (ARRIVE) guidelines and the European laws (Directive 2010/63/EU) that rule the use of animals in research.

### Metabolic measurements

Mice were weighed at 6 weeks and weekly thereafter. Blood glucose levels were measured using a Contour Next glucose meter (Bayer, Leverkusen, Germany). Food and caloric intake were also assessed. For glucose tolerance tests, mice were fasted overnight at 17 weeks of age, and blood glucose levels were measured before and 15, 30, 60, 90, and 120 min after intraperitoneal injection of glucose (2.0 g/kg). The area under the curve (AUC) was calculated using the trapezoidal rule for glucose data (16). For insulin tolerance tests, at 17 weeks of age, mice were fasted for 5 h, and blood glucose levels were measured before and 15, 30, 60, 90, and 120 min after intraperitoneal injection of human insulin (0.75 UI/Kg; Actrapid, Novo Nordisk).

### Insulin signaling studies

Mice were fasted overnight and intraperitoneally injected with human insulin (10 UI/kg of body weight; Actrapid, Novo Nordisk) or saline and sacrificed 10 min later. Organs [liver, epididymal white adipose tissue (WAT), interscapular brown adipose tissue (BAT), and gastrocnemius muscle] were harvested, snap frozen, and stored at −80°C until analysis. The liver was homogenized in lysis buffer (in mM: 20 Tris pH 7.5, 5 EDTA, 10 Na4P_2_O_7_, 100 NaF, 2 Na_3_VO_4_) with 1% NP-40 and protease inhibitors (Roche, Switzerland). Tissue lysates (20 µg protein) were mixed with Laemmli sample buffer (in %: 1.5 Tris, 20 glycerol, 4.1 SDS, 2 β-mercaptoethanol, 0.02 bromophenol blue, pH 6.8) and denatured for 10 min at 95°C. Proteins were fractionated in 10% polyacrylamide gels. The proteins were transferred from gels to previously activated PVDF membranes in TransBlot Turbo (Bio-Rad Laboratories, Hemel Hempstead, UK) and then blocked for 1 h in a 5% non-fat milk solution at room temperature. The membranes were incubated overnight at 4°C with the primary antibodies listed in **Supplementary Table S1** and incubated with secondary antibodies for 1 h. Either glyceraldehyde 3-phosphate dehydrogenase (GAPDH) or β-actin were used as loading controls. Membranes were reacted with Amersham ECL Prime Western Blotting Detection Reagent (GE Healthcare), read in Chemidoc, and quantified using ImageLab (Bio-Rad Laboratories).

### Liver histology and lipid assay

Liver was fixed with 10% formalin solution, embedded in paraffin, and cut in 4-μm sections. These sections were then stained with hematoxylin and eosin for the characterization of liver morphology and lipid content. Hepatic lipids were extracted as previously described (17). Briefly, approximately 250 mg of frozen tissue was rapidly mixed with high-performance liquid chromatography (HPLC)-grade methanol (4.6 ml/g) followed by methyl-tert-butyl ether (MTBE) (15.4 ml/g). The mixture was placed in a shaker for 4 h and then centrifuged at 13,000*g* for 10 min. The liquid fraction was collected, and phase separation was induced by adding 1 ml of distilled water and letting it rest at room temperature for 10 min. The liquid was then centrifuged for 10 min at 1,000*g*. The organic phase, containing the lipids, was separated and dried under nitrogen gas in a glass vial protected from light. It was then dissolved in butanol:(Triton X-100:methanol). Hepatic total cholesterol and triglyceride contents were determined by enzymatic method (SpinReact).

### Quantitative real-time polymerase chain reaction

The extraction of liver total ribonucleic acid (RNA) was performed using TRIzol (Invitrogen), and the concentrations were determined by a Nanodrop 2000 spectrophotometer (Thermo Fisher Scientific). Total RNA was reverse transcribed to complementary DNA (cDNA) using high-capacity cDNA reverse transcription kit (Applied Biosystems, CA, USA). Quantitative real-time polymerase chain reaction (qPCR) was performed to evaluate the abundance of messenger RNA (mRNA) coding for GK, G6Pase, PEPCK, ChREBP, SREBP2, ELOVL2, CD36, SCD1, β-actin, and β-2-microglobulin using SYBR™ Green PCR Master Mix (Applied Biosystems) in an ABI 7500 (Applied Biosystems). Specific exon–exon spanning primers were designed for the amplification of the target and housekeeping transcripts (**Supplementary Table S2**). β-Actin and β-2-microglobulin transcript levels were used to normalize gene expression levels. Fold variation in gene expression levels was calculated with the 2^−ΔΔCt^ method (18).

### Metabolite extraction

To extract the liver, gastrocnemius muscle, epididymal WAT, and interscapular BAT metabolites, the different tissues were homogenized in glass vials using a mixture of methanol and chloroform (2:1). After sonication on ice for 15 min, chloroform and water (1:1) were added, and samples were centrifuged at 10,000*g* for 15 min at 4°C. Polar and apolar fractions were isolated and evaporated using a flow of nitrogen. The polar fraction was dissolved in D_2_O phosphate buffer (0.2 M, pH 7) for proton nuclear magnetic resonance (^1^H-NMR) analysis.

### Proton nuclear magnetic resonance spectroscopy

Proton nuclear magnetic resonance (^1^H-NMR) spectra of the polar extracts were acquired using a Varian Inova 600 MHz (14.1 T) spectrometer equipped with a 3-mm QXI probe with a z-gradient. ^1^H-1D NOESY experiments with water presaturation were acquired at 298 K (7.2 kHz spectral width, 0.1 s mixing time, four dummy scans, 4 s relaxation delay with 3 s of water presaturation, 90°C pulse angle, 3 s acquisition time, and a minimum of 128 scans). For further spectral assignment, two-dimensional spectra (TOCSY) were acquired using sweep width of 5.4 kHz in both dimensions, 48 transients, and 400 and 1,024 points in t1 and t2 dimensions, respectively. Spectra were treated by multiplying FIDs with exponential window function (line broadening of 0.3 Hz) and were zero filled to 64 k points prior to Fourier transformation using TopSpin (Bruker Biospin, Karlsruhe, Germany). 2D spectra were processed by applying qsine window function and zero filled to 2,048 points in both dimensions. The comparison of 1D and 2D spectra with reference spectra and public databases such as HMDB allowed for peak assignment and metabolite identification (19). Metabolites were identified according to metabolomics standards initiative guidelines for metabolite identification (20).

### Multivariate analysis of NMR data

Processed 1D NOESY spectra were bucketed using one-point bucket (0.6–9.0 ppm, with signal-free, water, and fumarate regions excluded). Data matrix was built in Amix Viewer (BrukerBiospin, Rheinstetten). Icoshift algorithm (21) was used to align bucketed spectra, and total area integral normalization was applied to account for the variations in the overall sample concentrations. Multivariate statistical analysis was applied on unit variance scaled matrix (SIMCA 14, Umetrics, Sweden). Principal component analysis (PCA) was used to provide information on global data structure, and partial least squares discriminant analysis (PLS-DA) was used to assess group separation and to identify the main metabolites that contribute to the group discrimination. PLS-DA models were validated by sevenfold cross-validation and permutation test (n=100) to provide the qualitative measure of predictive power (Q2) and to assess the degree of fit to the data (R2). The corresponding PLS-DA loadings were obtained by multiplying the loading weight factors (w) by the standard deviation of the respective variable and were color-coded according to variable importance in the projection (VIP). All differentially expressed metabolites for each of the diets in each tissue were used to identify the most relevant metabolic pathways affected by diet using MetaboAnalyst (22).

### GC-MS analysis

Fatty acid methyl esters were obtained by base-catalyzed transmethylation (2 M KOH in methanol). The resultant fatty acid methyl ester solution was analyzed by gas chromatography using a Shimadzu GC-MS QP2010 UltraGas Chromatograph Mass Spectrometer (Shimadzu, Kyoto, Japan), with a capillary column BPX70 (0.25 mm internal diameter, 0.25 µm film thickness, 30 m long, SGE, Austin, TX, USA). Nonadecanoic fatty acid (C19:0) was used as the internal standard. The injector temperature was set at 250°CC, and 1 µl of each sample was injected with a split ratio of 1:80. Helium was used as the carrier gas. The initial column temperature was 155°CC, followed by a heating rate of 1°CC/min up to 170°CC, 4°CC/min up to 220°CC and 40°CC/min until reaching 250°CC, which was kept for 5 min. The linear velocity was 35 cm/s, with interface temperature of 250°CC, ion source temperature of 225°CC, mass range of 45–500, and event time of 0.3 s. All the measurements were repeated three times, and the average values were reported. Fatty acids were identified by retention time and fragmentation profile and quantified by the internal standard procedure. Results are expressed as a percentage of total fatty acids.

### Statistical analysis

Experimental data are shown as mean ± standard error of mean (SEM). Unless stated otherwise, statistical analysis was performed using one-way ANOVA in GraphPad Prism 8 (GraphPad Software, San Diego, CA, USA). p<0.05 was considered significant.

## Results

### HFat diet-fed mice develop glucose intolerance, insulin resistance, and non-alcoholic fatty liver disease

**Figure 1:**
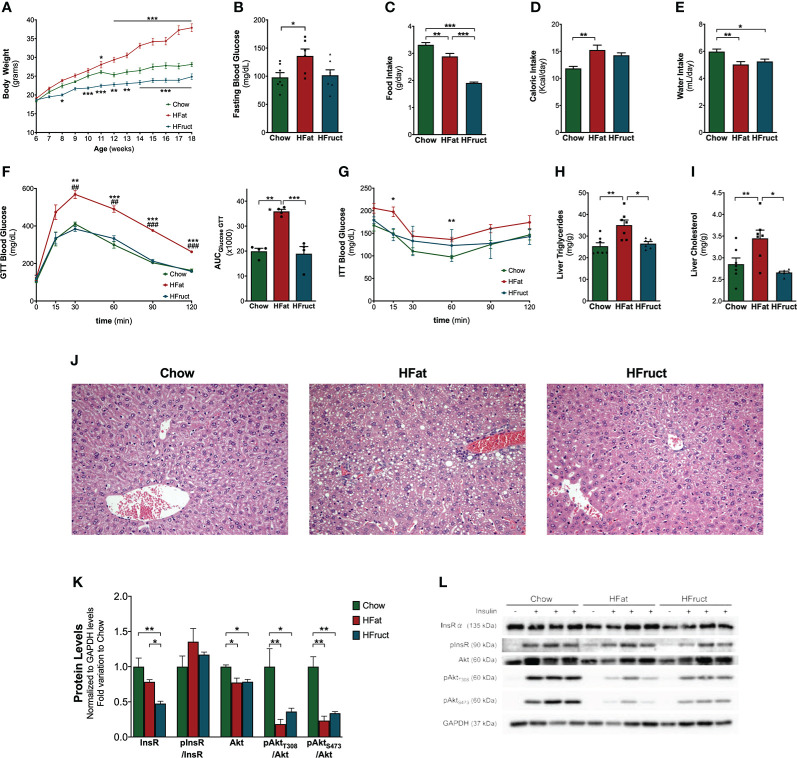
Effects of HFat and HFruct diets on metabolic parameters, glucose homeostasis, hepatic lipid deposition, and insulin signaling pathway in C57Bl/6J male mice. **(A)** Body weight, **(B)** intraperitoneal glucose tolerance test (ipGTT) and the respective area under the curve (AUC), **(C)** insulin tolerance test (ITT); **(D)** fasting blood glucose; **(E)** food intake; **(F)** water intake; **(G)** caloric intake; **(H)** hepatic triglycerides; **(I)** hepatic cholesterol levels; **(J)** representative images of hematoxylin and eosin-stained liver sections; **(K)** Chow, high-fat (HFat) and high-fructose (HFruct) hepatic protein levels of insulin receptor α (InsR), phosphorylated-insulin receptor (pInsR; normalized to InsR levels); protein kinase B (PKB/Akt) and its phosphorylation sites (pAkt_T308_ and pAkt_S473_; normalized to Akt levels) normalized to GAPDH levels and represented as fold variation to Chow group; **(L)** representative images of Western blot for the described antibodies. Data are presented as mean ± SEM for three to seven mice. *p<0.05, **p<0.01, ***p<0.001; **(F)** ^##^p<0.01 vs. HFruct, ^###^p<0.001 vs. HFruct.

Mice were subjected to a Chow, HFat, or HFruct diet. After a 12-week period of diet intervention, mice subjected to an HFat diet had increased body weight gain (37.90 ± 1.06 g) compared to Chow-diet-fed mice (28.14 ± 0.57 g; **Figure 1A**). Conversely, HFruct-diet-fed mice had decreased body weight (24.86 ± 0.76 g). In fact, after 12 weeks of diet, HFat-diet-fed mice had marked hyperglycemia (136 ± 12 mg/dl), compared to Chow-diet-fed mice (98 ± 8 mg/dl, **Figure 1B**), while HFruct-diet-fed mice were normoglycemic (102 ± 10 mg/dl). HFruct-diet-fed mice had decreased water intake, concomitant with decreased food intake, compared to Chow-diet-fed mice. However, this was not reflected in a decrease in caloric intake (**Figures 1C–E**). Caloric intake was increased by 25% in HFat-diet-fed mice, compared to Chow-diet-fed mice (**Figure 1D**). HFat-diet-fed mice were glucose intolerant, as observed by failure to decrease blood glucose levels after administration of a glucose bolus (**Figure 1F**). HFruct-diet-fed mice displayed normal glucose tolerance, but the ITT curve was flatter than Chow, revealing insulin resistance, as the capacity to decrease blood glucose levels after an insulin bolus is compromised (**Figures 1F, G**).

Dysmetabolism is associated with ectopic fat deposition in parenchymatous organs such as the liver. To evaluate this, hepatic triglycerides and cholesterol were measured. Liver content in both triglycerides and cholesterol was increased in HFat-diet-fed mice (35.1 ± 2.3 mg/g and 3.5 ± 0.2 mg/g, respectively) but unchanged in HFruct-diet-fed mice (26.5 ± 0.9 mg/g and 2.7 ± 0.03 mg/g, respectively; **Figures 1H, I**) when compared with Chow-diet-fed mice (25.4 ± 1.6 mg/g and 2.9 ± 0.1 mg/g, respectively). The analysis of histological sections demonstrated that HFat-diet-fed mice developed NAFL, i.e., presented lipid droplets in >5% of the sections, whereas HFruct-diet-fed mice had mild hepatic degeneration (**Figure 1J**). Insulin-stimulated glucose utilization relies on an effective insulin signaling and is known to impact the transcription of several lipid and carbohydrate metabolism-related genes (23). Therefore, the protein levels of the insulin pathway-related components, insulin receptor (InsR) and Akt, in the liver of the mice were determined. After 12 weeks of diet exposure, HFruct-diet-fed mice presented a decrease in InsRα levels. However, the activation of the pathway, observed by the ratio between phosphorylated-insulin receptor (pInsR) and InsR, did not present any differences between the groups, suggesting that InsR are functional (**Figures 1K, L**). When analyzing Akt phosphorylation levels, there was a marked reduction in both T308 and S473 in both HFat- and HFruct-diet-fed groups, revealing that both diets negatively impact on the activation of the insulin signaling pathway (**Figures 1K, L**).

### Hepatic carbohydrate and lipid and amino acid metabolisms are impaired by HFat and HFruct diets

**Figure 2:**
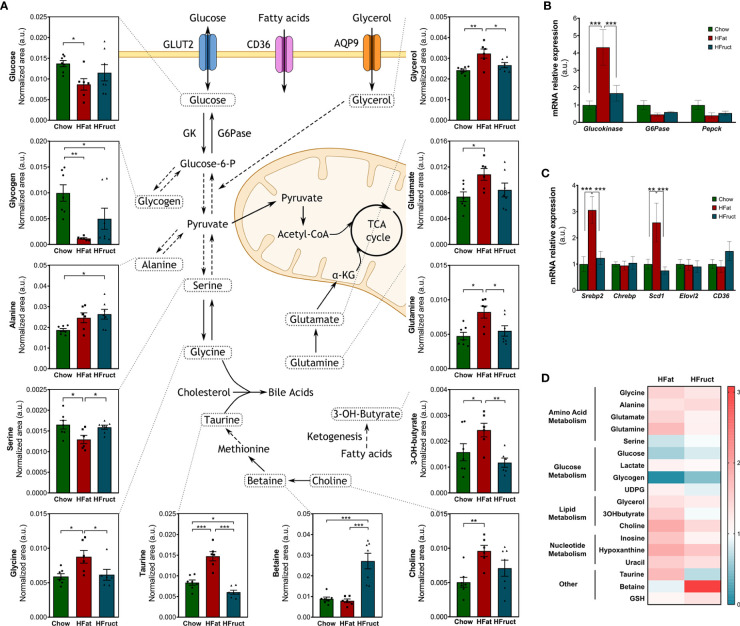
Effects of the HFat and HFruct diets on the hepatic metabolism of C57Bl/6J male mice. Chow, high-fat (HFat), and high-fructose (HFruct) hepatic levels of metabolites involved in glycolysis/gluconeogenesis, amino acid metabolism, ketogenesis, bile acid synthesis, and methionine cycle **(A)**, mRNA levels of glucose **(B)**, and lipid-related metabolism genes **(C)**, and heat map of hepatic polar metabolites in comparison to Chow metabolites, which are represented by 1/white **(D)**. α-KG, α-ketoglutarate; AQP9, aquaporin 9; CD36, cluster of differentiation 36; G6Pase, glucose-6-phosphatase; GK, glucokinase; GSH, glutathione; TCA, tricarboxylic acid; UDPG, uridine diphosphate glucose. Data are presented as mean ± SEM for five to seven mice. *p<0.05, **p<0.01; ***p<0.001.

The liver is a key organ for the maintenance of normal glucose homeostasis. However, this homeostasis is impaired in insulin-resistant states; thus, we evaluated the intracellular levels of key metabolites in the fasting state, and the mRNA expression of some glucose metabolism-related genes. Intra-hepatic glucose levels were decreased in HFat-diet-fed mice compared to Chow-diet-fed mice (**Figure 2A**). The mRNA levels of glucokinase, the enzyme responsible for the conversion of glucose into glucose-6-phosphate, thus enabling the trapping of glucose inside the cells, are increased in HFat-diet-fed mice (4.32 ± 1.02-fold variation to Chow), when compared to both Chow- and HFruct-diet-fed mice (1.00 ± 0.24- and 1.68 ± 0.45-fold variation to Chow, respectively; **Figure 2B**). The mRNA expression of glucose-6-phosphatase, the enzyme that converts glucose-6-phosphate to glucose, does not present any significant difference between the groups, although there is a decrease in the HFat group, when compared to Chow (0.45 ± 0.09- and 1.00 ± 0.26-fold variation to Chow, respectively; p=0.06; **Figure 2B**). The expression of *Pepck*, responsible for glucose production from pyruvate, was also analyzed, although no statistical differences were found. In the fasting state, the liver produces glucose from several substrates, namely, glycogen. However, we observed that both HFat- and HFruct-diet-fed mice present lower levels of glycogen in the liver when compared to Chow-diet-fed mice. Glucose may also enter glycolysis to yield pyruvate; the latter has three major outcomes, namely, acetyl-CoA, acetate, or lactate. However, we observed that hepatic lactate and acetate levels do not vary significantly with the diet (data not shown).

Like for carbohydrates, the liver is essential for fatty acid metabolism. Dysregulations in cholesterol biosynthesis or *de novo* lipogenesis, in a key lipogenic tissue as the liver, may disrupt the overall lipid homeostasis. Thus, we evaluated intracellular levels of key metabolites for lipid metabolism and the mRNA expression of some lipid-metabolism-related genes. No statistical differences were found regarding *CD36* mRNA levels nor *Elovl2*, responsible for the elongation. However, *Srebp2* and *Scd1* mRNA levels were increased in HFat-diet-fed mice (3.06 ± 0.51- and 2.59 ± 0.74-fold variation to Chow; **Figure 2C**), when compared to both Chow (1.00 ± 0.29- and 1.00 ± 0.19-fold variation to Chow) and HFruct groups (1.23 ± 0.26- and 0.76 ± 0.14-fold variation to Chow; **Figure 2C**). Glycerol, a product of adipose tissue lipolysis, is an important substrate for both gluconeogenesis and lipogenesis (24). Glycerol levels were increased in the liver of HFat-diet-fed mice, when compared to Chow-diet-fed mice. Choline, which is the precursor for the essential component of the very low density lipoprotein (VLDL) phosphatidylcholine, was increased in HFat-diet-fed mice, compared to Chow-diet-fed mice (**Figure 2A**). Choline is oxidized to betaine, an osmoregulator and a methyl-group donor (25). Betaine was increased in HFruct-fed-mice compared to both Chow- and HFat-fed mice (**Figure 2A**). When glycogen is not available to produce glucose as energy substrate, ketogenesis takes place. One of the metabolites produced during this process is 3-hydroxybutyrate, which was increased in HFat-diet-fed mice, when compared to Chow- and HFruct-diet-fed animals (**Figure 2A**).

Besides being the basic units for protein synthesis, amino acids serve as intermediate metabolites for TCA cycle and lipid and nucleotide biosynthesis and sustain cell proliferation. Amino acid levels were significantly altered in the liver tissue by both high-fat and high-fructose diets. Serine, besides being a precursor of proteins and lipids, is involved in glycogen storage in the liver (26). Its levels were decreased in HFat-diet-fed mice, when compared to both Chow- and HFruct-diet-fed animals (**Figure 2A**). Serine is inter-convertible with glycine (27). The levels of this amino acid were increased in ~50% in the HFat group, when compared with both Chow- and HFruct-diet-fed mice (**Figure 2A**). During fasting, alanine participates in gluconeogenesis. Alanine levels were increased in almost 40% in HFruct mice when compared to Chow-diet-fed mice (**Figure 2A**). α-Ketoglutarate (α-KG), one of the metabolites of the Krebs cycle, may be interconverted into glutamate, which may be, in turn, interconverted in glutamine, an important amino acid and a precursor for gluconeogenesis and glutathione synthesis (28). Both glutamate and glutamine were increased in HFat-diet-fed mice when compared to Chow-diet-fed animals (**Figure 2A**). A graphical diagram with the most affected metabolites is depicted in **Figure 2A**, along with the graphs for each metabolite and a heat map (**Figure 2D**); the most affected pathways in the liver by HFat or HFruct feeding is are shown in **Supplementary Figures 1A, B**, respectively.

### HFat and HFruct diets increase hepatic monounsaturated fatty acids and decrease n-6 polyunsaturated fatty acids

As previously mentioned, the liver is a key lipogenic tissue; thus, fatty acid methyl esters levels were analyzed upon HFat and HFruct feeding for 12 weeks. After grouping fatty acids by saturation degrees, we observed that HFat diet leads to an increase in relative abundance of monounsaturated fatty acids (MUFA; 45.60 ± 1.61) when compared to Chow-fed mice (27.91 ± 1.13; **Table 1**). Moreover, HFat-fed mice present decreased relative abundance of n-3 and n-6 polyunsaturated fatty acids (PUFAs), when compared to Chow-fed mice and HFruct-fed mice (**Table 1**). The decrease in n-3 PUFA is mainly attributed to a decrease in docosahexaenoic acid (DHA; C22:6 n3) and α-linolenic acid (C18:3n3), while the decrease in n-6 PUFA is mainly due to a decrease in linoleic acid (C18:2n6).

**Table 1:** Fatty acid composition of liver tissue obtained from normal chow diet (Chow), high-fat diet (HFat), and high-fructose (HFruct)-fed male C57Bl6/J mice after 12 weeks of diet.

Fatty Acid	Chow	HFat	HFruct
**C10:0**	0.000 ± 0.000	0.017 ± 0.003	0.000 ± 0.000
**C12:0**	0.141 ± 0.027	1.370 ± 0.225	0.159 ± 0.028
**C14:0**	0.517 ± 0.051	3.182 ± 0.272^a^	0.494 ± 0.047^b^
**C15:0**	0.090 ± 0.005	0.082 ± 0.005	0.081 ± 0.005
**C16:0**	24.097 ± 0.309	25.915 ± 0.225^a^	20.880 ± 0.318^a,b^
**C16:1n7**	2.876 ± 0.256	8.025 ± 0.280^a^	3.004 ± 0.197^b^
**C18:0**	6.801 ± 0.538	4.027 ± 0.272^a^	7.793 ± 0.562^b^
**C18:1n9**	22.877 ± 0.889	33.708 ± 1.234^a^	29.316 ± 0.961^a,b^
**C18:1n7**	1.917 ± 0.045	5.440 ± 0.541^a^	2.570 ± 0.047^b^
**C18:2n6**	25.679 ± 1.135	8.312 ± 0.613^a^	19.856 ± 0.312^a,b^
**C18:3n3**	0.623 ± 0.063	0.208 ± 0.021	0.414 ± 0.017
**C20:1n9**	0.236 ± 0.033	0.430 ± 0.036	0.379 ± 0.023
**C20:3n6**	0.497 ± 0.053	0.503 ± 0.020	0.843 ± 0.069
**C20:4n6**	8.167 ± 0.764	4.845 ± 0.335^a^	8.260 ± 0.474^b^
**C22:4n6**	0.197 ± 0.012	0.152 ± 0.009	0.167 ± 0.010
**C22:5n6**	0.121 ± 0.014	0.263 ± 0.015	0.124 ± 0.009
**C22:5n3**	0.299 ± 0.012	0.227 ± 0.025	0.256 ± 0.006
**C22:6n3**	4.863 ± 0.425	3.312 ± 0.244^a^	5.407 ± 0.298^b^
**∑ MUFA**	27.906 ± 1.134	47.597 ± 1.609^a^	35.270 ± 1.118 ^a,b^
**∑ n3 PUFA**	5.783 ± 1.134	3.745 ± 0.279^a^	6.074 ± 0.289^b^
**∑ n6 PUFA**	34.661 ± 0.958	14.074 ± 0.895^a^	29.274 ± 0.435^a,b^
**∑ SFA**	31.650 ± 0.622	34.585 ± 0.625^a^	29.407 ± 0.560^a,b^

Values represent mean ± SEM for five to seven mice per experimental group. ^a^p<0.05 vs. Chow; ^b^p<0.05 vs. HFat. Monounsaturated fatty acids (MUFAs) correspond to C16:1n7, C18:1n9, C18:1n7, and C20:1n9. n-3 polyunsaturated fatty acids (PUFAs) correspond to C18:3n3, C22:5n3, and C22:6n3; n-6 PUFA correspond to C18:2n6, C20:3n6, C20:4n6, C22:4n6, and C22:5n6. Saturated fatty acids (SFAs) correspond to C10:0, C12:0, C14:0, C15:0, C16:0, and C18:0.

### High-fat and high-fructose diets differentially affect muscle metabolome

**Figure 3:**
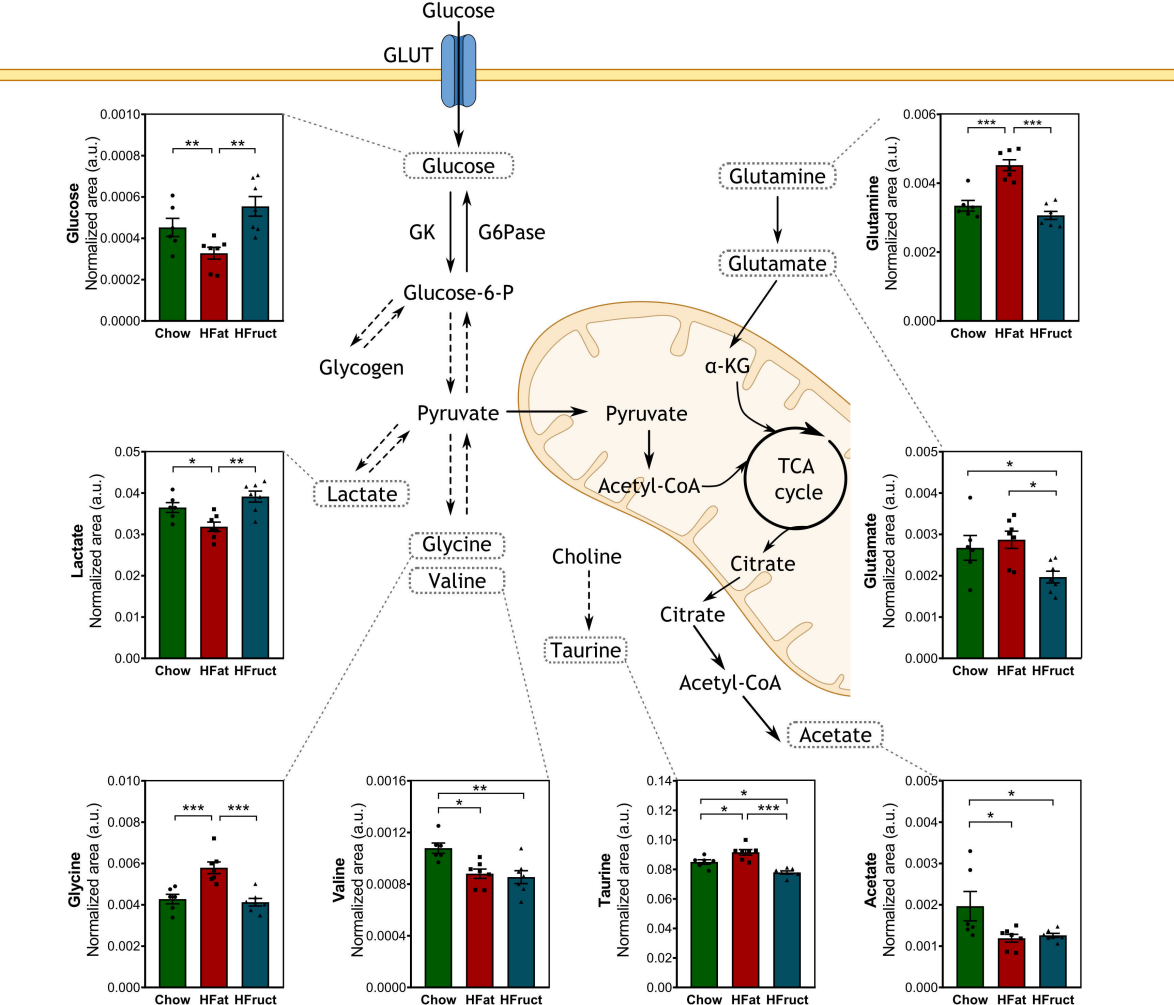
Effects of the HFat and HFruct diets on the muscle metabolism of C57Bl/6J male mice. Chow, high-fat (HFat), and high-fructose (HFruct) muscle levels of metabolites involved in glycolysis/gluconeogenesis and amino acid metabolism. α-KG, α-ketoglutarate; G6Pase, glucose-6-phosphatase; GK, glucokinase; GLUT, glucose transporter; IMP, inosine monophosphate; TCA, tricarboxylic acid. Data are presented as mean ± SEM for five to seven mice. *p<0.05, **p<0.01; ***p<0.001.

Skeletal muscle is a major organ of glucose uptake, storage, and usage. The muscle can store glucose in the form of glycogen, which is crucial for the rapid initiation of energy production even when glucose is not readily available (29). Therefore, we evaluated the muscle intracellular polar metabolites after 12 weeks of HFat or HFruct feeding. Muscular glucose levels were decreased in HFat-diet fed mice, compared with both Chow and HFruct-diet-fed mice (**Figure 2A**). Lactate, which may be converted into pyruvate, was also decreased in HFat-diet-fed mice compared with both Chow- and HFruct-diet-fed mice. In the need of substrate, fatty acids stored as triglycerides or amino acids may be metabolized (30). In fact, dietary changes had the major impact in amino acids levels. Glycine and glutamine were increased in HFat-diet-fed mice compared to the other diets, which may promote and contribute for protein synthesis (31). Both dietary interventions caused a decrease in valine, whose metabolism produces ammonia by-products that may participate in the conversion of glutamate to glutamine (32), but only HFruct led to decreased levels of glutamate, when compared to Chow-diet-fed mice. A graphical diagram with the most affected metabolites is depicted in **Figure 3**, along with the graphs for each metabolite, and a heat map is depicted in **Supplementary Figure 2A**, and the most affected pathways in the muscle by HFat (**Supplementary Figure 2B**) or HFruct feeding (**Supplementary Figure 2C**).

### Epidydimal white adipose tissue metabolome is less affected by high-fat and high-fructose feeding than the liver and muscle

**Figure 4:**
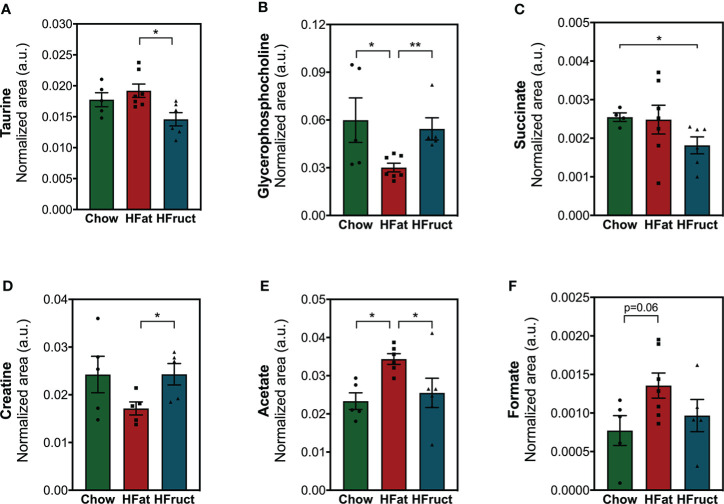
Effects of the HFat and HFruct diets on the epididymal white adipose tissue (WAT) metabolism of C57Bl/6J male mice. **(A)** Taurine; **(B)** glycerophosphocholine; **(C)** succinate; **(D)** creatine; **(E)** acetate; **(F)** formate relative areas in WAT after 12 weeks of normal chow (Chow), high-fat (HFat), or high-fructose (HFruct) feeding. Data are presented as mean ± SEM for four to seven mice. *p<0.05, **p<0.01.

As a source of energy substrates, WAT responds to variations in the body’s nutritional status and energy demand (33). Herein, we observed that HFat feeding for 12 weeks led to increased acetate levels (**Figure 4E**), which might indicate suppressed lipolysis (34), and decreased glycerophosphocholine (**Figure 4B**) when compared to Chow. Moreover, HFat-fed mice presented decreased creatine, a metabolite known for stimulating energy expenditure (35) (**Figure 4D**), and increased taurine (**Figure 4A**), when compared to HFruct-fed mice, all pointing towards an obesogenic mechanism (36). On the other hand, HFruct animals had decreased succinate, when compared to Chow-fed mice (**Figure 4C**). No significant differences were observed in formate levels (**Figure 4F**). A heat map with analyzed metabolites is depicted in **Supplementary Figure 3A** and the most affected pathways in WAT by HFat (**Supplementary Figure 3B**).

### Brown adipose tissue metabolome is modulated by high-fat or high-fructose feeding

**Figure 5:**
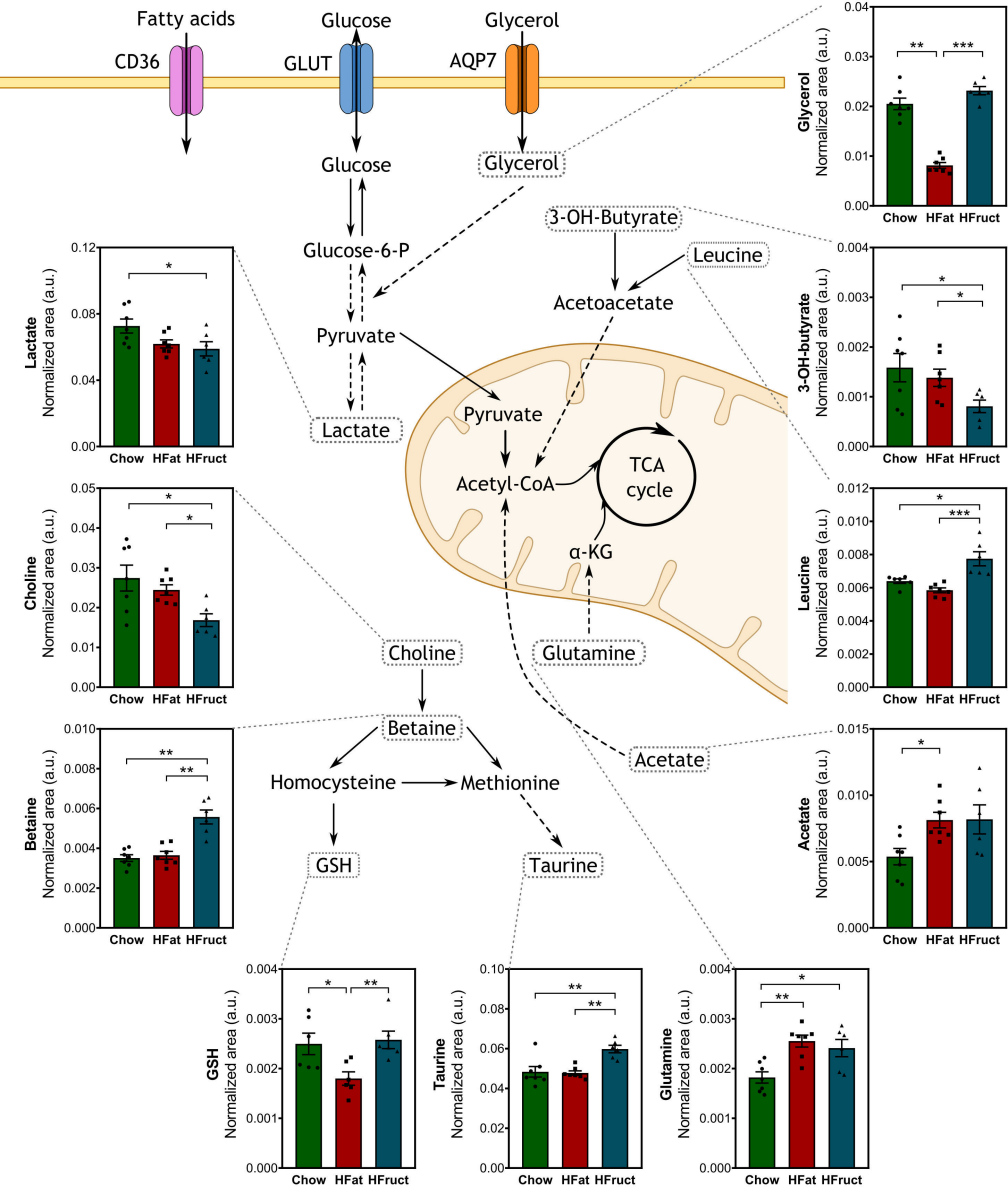
Effects of the HFat and HFruct diets on the brown adipose tissue (BAT) metabolism of C57Bl/6J male mice. Chow, high-fat (HFat), and high-fructose (HFruct) BAT levels of metabolites involved in glycolysis/gluconeogenesis, amino acid metabolism, and methionine cycle. α-KG, α-ketoglutarate; AQP7, aquaporin 7; CD36, cluster of differentiation 36; G6Pase, glucose-6-phosphatase; GK, glucokinase; GLUT, glucose transporter; GSH, glutathione; IMP, inosine monophosphate; TCA, tricarboxylic acid. Data are presented as mean ± SEM for five to seven mice. *p<0.05, **p<0.01; ***p<0.001.

Although it has been disregarded for years, studies about the presence of BAT in adults and the discovery of its crosstalk with important metabolic organs, namely, the muscle, has brought BAT to the spotlight (37). The BAT metabolome was assessed, and the graphical diagram in **Figure 5** discloses the most affected metabolites, along with the graphs for each metabolite, and a heat map is depicted in **Supplementary Figure 4A** and the most affected pathways in BAT by HFat (**Supplementary Figure 4B**) or HFruct feeding (**Supplementary Figure 4C**). HFruct led to an increase in betaine, taurine, glutamine, and leucine. In contrast, the same diet led to a decrease in 3-hydroxybutyrate when compared to Chow-diet-fed mice (**Figure 5**). On the other hand, HFat diet led to a decrease in glutathione (GSH), which was already reported to be inversely correlated with the activation of thermogenesis, the hallmark function of BAT (38). Moreover, HFat feeding resulted in decreased levels of glycerol, when compared to Chow-diet-fed mice. On the other hand, HFat caused an increase in glutamine and acetate compared with Chow diet, and the latter was already associated with upregulation of mitochondrial biogenesis, contributing to increased thermogenesis (39).

### HFat decreases n-6 PUFA while HFruct diets increase MUFA in brown adipose tissue

BAT fatty acid methyl esters levels were measured upon HFat and HFruct feeding for 12 weeks. We observed that HFat diet leads to an increase in relative abundance of saturated fatty acids (SFAs; 44.27 ± 0.45) when compared to Chow-fed mice and HFruct-fed mice (25.58 ± 0.75 and 23.62 ± 0.96, respectively; **Table 2**). On the contrary, n-6 PUFA were decreased in HFat (7.95 ± 0.13) fed mice, when compared to both Chow- (24.65 ± 1.72) and HFruct (19.63 ± 0.42)-fed mice, mainly due to a decrease in linoleic acid (C18:2n6). MUFA were increased in HFruct-fed mice (56.10 ± 0.82) in comparison with both Chow- (48.82 ± 1.11) and HFat (47.45 ± 0.48)-fed mice, reflecting an increase in elaidic acid (C18:1n9).

**Table 2:** Fatty acid composition of brown adipose tissue (BAT) obtained from normal chow diet (Chow), high-fat diet (HFat), and high-fructose (HFruct)-fed male C57Bl6/J mice after 12 weeks of diet.

Fatty Acid	Chow	HFat	HFruct
**C10:0**	0.013 ± 0.005	0.210 ± 0.013	0.023 ± 0.012
**C12:0**	0.390 ± 0.155	9.767 ± 0.330^a^	1.010 ± 0.384^b^
**C14:0**	1.896 ± 0.137	11.261 ± 0.141^a^	2.137 ± 0.358^b^
**C15:0**	0.074 ± 0.004	0.066 ± 0.003	0.046 ± 0.008
**C16:0**	18.601 ± 0.761	19.777 ± 0.134^a^	14.823 ± 0.461^a,b^
**C16:1n7**	5.709 ± 0.560	9.066 ± 0.279^a^	4.369 ± 0.213^a,b^
**C18:0**	4.314 ± 0.100	2.947 ± 0.111^a^	4.804 ± 0.158^b^
**C18:1n9**	39.277 ± 0.550	35.859 ± 0.474^a^	47.856 ± 0.854^a,b^
**C18:1n7**	2.976 ± 0.165	2.109 ± 0.049	2.853 ± 0.088
**C18:2n6**	23.689 ± 1.650	7.397 ± 0.121^a^	18.016 ± 0.403^a,b^
**C18:3n3**	0.569 ± 0.027	0.211 ± 0.006	0.326 ± 0.019
**C20:0**	0.256 ± 0.028	0.239 ± 0.019	0.727 ± 0.053
**C20:1n9**	0.861 ± 0.041	0.417 ± 0.013	1.027 ± 0.042
**C20:3n6**	0.184 ± 0.008	0.084 ± 0.003	0.186 ± 0.009
**C20:4n6**	0.703± 0.060	0.433 ± 0.025	1.369 ± 0.104
**C22:0**	0.030 ± 0.009	0.000 ± 0.000	0.050 ± 0.012
**C22:4n6**	0.051 ± 0.010	0.019 ± 0.003	0.036 ± 0.007
**C22:5n6**	0.024 ± 0.005	0.020 ± 0.004	0.023 ± 0.004
**C22:5n3**	0.046 ± 0.009	0.024 ± 0.003	0.027 ± 0.005
**C22:6n3**	0.336 ± 0.023	0.097 ± 0.006	0.297 ± 0.009
**∑ MUFA**	48.823 ± 1.112	47.449 ± 0.479	56.104 ± 0.823^a,b^
**∑ n3 PUFA**	0.950 ± 0.047	0.334 ± 0.005^a^	0.649 ± 0.018^a,b^
**∑ n6 PUFA**	24.650 ± 1.719	7.951 ± 0.134^a^	19.630 ± 0.424^a,b^
**∑ SFA**	25.577 ± 0.750	44.267 ± 0.447^a^	23.617 ± 0.961^b^

Values represent mean ± SEM for five to seven mice per experimental group. ^a^p<0.05 vs. Chow; ^b^p<0.05 vs. HFat. Monounsaturated fatty acids (MUFAs) correspond to C16:1n7, C18:1n9, C18:1n7, and C20:1n9. n-3 polyunsaturated fatty acids (PUFA) correspond to C18:3n3, C22:5n3, and C22:6n3; n-6 PUFA correspond to C18:2n6, C20:3n6, C20:4n6, C22:4n6, and C22:5n6. Saturated fatty acids (SFAs) correspond to C10:0, C12:0, C14:0, C15:0, C16:0, C18:0, C20:0, and C22:0.

## Discussion

The sedentary lifestyle and poor nutritional habits have led to an increase in the prevalence of metabolic disorders, such as obesity, T2DM and NAFLD. Importantly, the increased consumption of ultra-processed food resulted in increased intake of simple carbohydrates and saturated fat (40). Although it is already known that the enrichment in lipids and in carbohydrates, particularly fructose, has different pathophysiological effects, the consequence of these specific components of the diet on the metabolome of important metabolic organs such as the liver, muscle, and adipose tissue depots remains to be fully understood.

HFat diet, alone or combined with sucrose as is the case of the present study (D12331, ResearchDiets), is known to lead to obesity and insulin resistance in mice, originating a similar metabolic imbalance to what is observed in humans (41–43). On the other hand, fructose alone does not cause a significant increase in body weight (44). This effect is also seen in humans, and, in comparison with glucose, fructose has less effects on body weight gain (45), despite fructose deleterious effects in inflammatory processes (46).

The current study confirmed that, at 18 weeks of age and after 12 weeks of feeding, HFat-diet-fed mice had higher caloric intake, body weight, and hyperglycemia than Chow-diet-fed mice. Moreover, HFat-diet-fed mice were glucose intolerant and insulin resistant. Conversely, HFruct-diet-fed mice presented lower body weight gain than the Chow group and normal glucose tolerance. Although these animals had a decreased food intake, compared to the Chow group, the caloric intake was similar. Both food, water, and caloric intake results are supported by previous studies, although with a slightly different percentage of fructose (44, 47).

Nutritional imbalances promoting ectopic lipid accumulation are particularly striking in the liver (48, 49). Our results are in line with what is already described in the literature (50, 51). Indeed, HFat mice developed NAFL, with increased triglycerides and cholesterol. HFruct-diet-fed mice developed mild hepatic degeneration mainly in periportal areas. This impact of an HFruct diet on liver histology may be due to the activation of a lipogenic pathway and the absence of a regulatory mechanism of fructose metabolism, contrarily to what happens with glucose (52, 53). Consequently, fructolysis will continue to occur, even when glycolysis is inhibited due to positive cellular energy balance. In fact, this effect was already been observed in studies with as low as 10% of fructose in drinking water (50). A proper glucose usage depends on a functional insulin signaling pathway. Indeed, hepatic insulin signaling was severely affected by both diets. A profound decrease in Akt phosphorylation at both phosphorylation sites was found, demonstrating that these animals are insulin resistant due to an impairment of the insulin signaling pathway, upstream of Akt, but downstream of the InsR (54). These results are supported by previous studies where insulin signaling is severely affected by fructose intake, namely, through a decrease in Akt phosphorylation (53, 55).

The liver is the most important metabolic organ, regulating carbohydrate, lipid, and protein metabolism (56). While in the fed state, liver takes up glucose that is either stored as glycogen or converted into fatty acids; in the fasted state, liver produces and releases glucose through glycogenolysis and gluconeogenesis (56). Subsequently, substrates as glucose and triglycerides go into the bloodstream and are metabolized by peripheral organs. Adipose tissue, in turn, releases non-esterified fatty acids and glycerol. Together with alanine and lactate, which are released by the muscle, these metabolites are used as precursors for gluconeogenesis. Hepatic glycogen levels were decreased in HFat-diet-fed mice. Interestingly, glucokinase expression levels were increased in the liver, while G6Pase levels were unchanged. These results point towards a metabolic dysregulation; as in the fasted state, it is expected that glucokinase transcription decreases in favor of G6Pase expression. In addition, glycerate-3-phosphate produced during glycolysis can be converted to serine, which is interconvertible with glycine. While serine was decreased in the liver of HFat-diet-fed mice, glycine was increased. In fact, decreased hepatic levels of serine, due to a downregulation of phosphoglycerate dehydrogenase, contribute to the development of fatty liver disease (57). One of the pathological features of fatty liver disease is hepatocyte swelling, which was observed in our study in HFat-diet-fed mice. Indeed, glutamine, a potent osmoregulator that contributes to cell swelling (28), was increased in HFat-diet-fed mice compared to Chow-fed mice. Moreover, glutamine, is known for promoting stimulation of canalicular bile salt excretion (58). On the other hand, glycine is involved in the enterohepatic cycle of bile acids, key players in lipid absorption and regulation of cholesterol homeostasis (27), which were also increased in the HFat group. Aside from glycine, taurine, which is also conjugated with cholesterol for the synthesis of bile acids, was increased in the liver of HFat-diet-fed mice. All these results point towards an increased production of bile acids. In fact, bile acids are crucial for an effective digestion of lipids (59). As these amino acids and cholesterol were increased only in HFat-diet-fed mice, it suggests a possible response to the increased lipid intake. Consistently, we also observe an increase in hepatic Srebp2 and Scd1 expression, suggesting that the lipogenic pathway may be active as a response to increased levels of hepatic fatty acid levels. Moreover, glycerol was increased in the liver of HFat-diet-fed mice, pointing towards an increase in adipose tissue lipolysis, which goes in line with previous studies in adipocytes (60). Additionally, dietary composition had repercussions on hepatic lipid composition, and we observed an increase in hepatic SFA in HFat-fed mice compared to Chow. SFAs are potent lipotoxic species and induce apoptosis and proinflammatory pathways through several mechanisms (61). Although this increase was not so prominent, there was also a decrease in PUFA, known to play a protective role against cell injury by clearing fat from hepatocytes and improving liver histology and lipid profile in patients with NASH (62). Altogether, this imbalance in the lipid species might contribute to NAFL development in the HFat group. On the other hand, the HFruct group also presents a decrease in hepatic glycogen levels, without changes in the expression of any of the analyzed glucose- and lipid-metabolism-related genes. However, there was an increase in MUFA and a decrease in n-6 PUFA in comparison with Chow. In fact, there have been contradictory results in the literature regarding MUFA levels and Scd1 mRNA expression (63); however, the animals were fed with 60% (w/v) fructose for a longer period than in the present study, which indicates that the effects of fructose on the activation of lipogenic pathways are time and dose dependent. Interestingly, we found an increase in alanine levels, in concurrence with the data obtained in a recent study with HFruct-fed rats (64). This increase in alanine levels may be a consequence of an increase in the release of alanine from the muscle that is then delivered to the liver through Cahill cycle or to its conversion from pyruvate in the liver.

The skeletal muscle is a major site for glucose metabolism. Insulin resistance in the muscle is known to cause glycogen depletion, lipid accumulation, impairment of the tissue’s normal functions, ultimately leading to sarcopenia (29). Muscle stores glucose in the form of glycogen, which facilitates the rapid initiation of energy production for contraction, even when glucose is not readily available from circulation (29). In this study, HFat diet promoted a decrease in glucose and lactate pools in the muscle, pointing towards the use of other substrates to obtain energy. In the need for other substrates, amino acids may be metabolized. Skeletal muscles are the most relevant site of glutamine stock, synthesis, and release (65). In this study, glutamine levels were increased in HFat-fed mice, and glutamate levels remained similar to the levels detected in the muscle of Chow-diet-fed mice. Glutamate levels can be used to produce glutamine that will be then delivered to the liver where it may have several functions (65, 66). Indeed, and as there was no glycogen pool in the liver, gluconeogenesis may use glutamine as a substrate for glucose production after an overnight fasting (32), as was the case of the present study. On the contrary, HFruct diet promoted a decrease in muscular glutamate levels, which might negatively affect glutathione synthesis and/or purine nucleotide cycle (67). Moreover, a decrease in valine was observed in both HFat- and HFruct-fed groups. Valine is used to obtain energy with the resulting ammonia by-products participating in the conversion of glutamate to glutamine, thus sustaining the previously mentioned hypothesis. Although it has already been described that diet-induced obese animals have a decrease in branched-chain amino acids (BCAAs) (68), there are studies describing a positive correlation between valine circulating levels and insulin resistance in men (69, 70).

WAT has a crucial role for lipid homeostasis, namely, by regulating triglyceride levels, and for fatty acid availability by lipolysis, also generating substrates for energy metabolism *via* β‐oxidation. In fact, we observed an increase in the WAT levels of acetate in HFat-fed mice compared to Chow, which indicates increased β‐oxidation (71). On the contrary, creatine was decreased in HFat-fed mice compared to HFruct-fed mice. As this metabolite enhances energy expenditure (35), the finding goes in accordance with the larger fat depots in this group. Although taurine promotes thermogenesis in the BAT and muscle, it does not have the same effect in WAT (72), which was also not observed in the present study. In fact, taurine supplementation was shown to prevent obesity in female mice after 18 weeks of high-fat feeding (36). In this study, male mice fed with HFat diet for 12 weeks presented no differences in taurine compared to the Chow group, which might indicate that decreases in taurine may be more noticeable in females or only appear with prolonged HFat feeding.

Organ crosstalk is mediated by signaling factors and ensures whole-body metabolic homeostasis. However, in case of disease or metabolic dysfunction, metabolic impairment in one organ will lead to dysregulation of others. Although this is known, the interplay and metabolic crosstalk between the different organs remain obscure. In recent years, BAT was rediscovered, and very recently, the existence of a crosstalk with the liver and the muscle was unveiled (73). HFat diet led to a decrease in GSH levels in the BAT compared with both Chow and HFruct diets. GSH was already reported to be inversely correlated with the activation of thermogenesis in white adipocytes due to its action on forkhead box O1 (74). Consequently, decreased GSH levels might have the same effect in BAT and increase thermogenesis in the BAT of HFat-diet-fed mice (38). Moreover, HFat-diet-fed mice had increased levels of glutamine and acetate in BAT. Acetate upregulates mitochondrial biogenesis, which is the key organelle for thermogenesis (39). Importantly, HFruct feeding led to an increase in betaine and taurine, probably at the expense of decreased choline. In fact, betaine protects against fructose-induced inflammation in astrocytes (75). HFruct-diet-fed animals also presented increased leucine. This amino acid is an activator of the mammalian target of rapamycin (mTOR) pathway, leading to reduced thermogenesis (76, 77). Moreover, the levels of 3-OH-butyrate, considered an alternative carbon source for thermogenesis (78), were decreased in HFruct-fed mice. Thus, high-fat feeding seems to be an activator of thermogenesis, while HFruct leads to a decrease in this process. Moreover, it was already reported that in HFat conditions, BAT functions largely like WAT in its role as a depot for excess energy (79). In these cases, adipocytes suffer a remodeling due to its storage needs and, consequently, the need of the membrane to be more fluid (80). In fact, we observed a completely different fatty acid distribution in HFat-fed mice compared to both Chow- and HFruct-fed mice that were lean. This difference might represent the need of the adipocytes to remodel and be able to store more fat.

In summary, our data provide novel evidence that, aside from the extensively described effects of high-fat and high-fructose diets on glucose and insulin homeostasis, these diets differentially and significantly affect liver, muscle, WAT, and BAT metabolic profiles. Interestingly, our data suggests a crosstalk between the liver, muscle, and BAT, specifically through alanine, glutamine, and lactate.

## Data availability statement

The raw data supporting the conclusions of this article will be made available by the authors, without undue reservation.

## Ethics statement

The animal study was reviewed and approved by DGAV - Portuguese General Directorate of Food and Veterinary Medicine and by NOVA Medical School Ethics Committee.

## Author contributions

MJM: experimental work, animal studies, data analysis and interpretation, and writing of the manuscript. IS-L: experimental work, animal studies, and data analysis and interpretation. IJ: NMR and data analysis. JFR: data interpretation. MGA: conceptualization, data interpretation, and writing of manuscript. MPM: conceptualization, supervision, data interpretation, and writing of the manuscript. All authors read and agree with the final version of the manuscript.

## Funding

This work was supported by “Fundação para a Ciência e a Tecnologia”—FCT MJM (PD/BD/114256/2016), MPM (PTDC/BIM-MET/4265/2014 and PTDC/MEC-MET/29314/2017), MGA (PTDC/BIM-MET/4712/2014), iNOVA4Health (UIDB/Multi/04462/2020), by the European Commission Marie Skłodowska-Curie Action H2020 (mtFOIE GRAS, grant agreement n. 734719), by the Sociedade Portuguesa de Diabetologia, and by the research infrastructure CONGENTO, project LISBOA-01-0145-FEDER-022170, co-financed by Lisboa Regional Operational Programme (Lisboa2020), under the PORTUGAL 2020 Partnership Agreement, through the European Regional Development Fund and by Fundação para a Ciência e Tecnologia (Portugal). NMR data were collected at the UC-NMR facility which is supported in part by FEDER – European Regional Development Fund through the COMPETE Programme (Operational Programme for Competitiveness) and by National Funds through FCT – Fundação para a Ciência e a Tecnologia (Portuguese Foundation for Science and Technology) through grants REEQ/481/QUI/2006, RECI/QEQ-QFI/0168/2012, CENTRO-07-CT62-FEDER-002012, and Rede Nacional de Ressonância Magnética Nuclear (RNRMN).

## Conflict of interest

The authors declare that the research was conducted in the absence of any commercial or financial relationships that could be construed as a potential conflict of interest.

The reviewer RS declared a shared affiliation with the author MA to the handling editor at the time of review.

## Publisher’s note

All claims expressed in this article are solely those of the authors and do not necessarily represent those of their affiliated organizations, or those of the publisher, the editors and the reviewers. Any product that may be evaluated in this article, or claim that may be made by its manufacturer, is not guaranteed or endorsed by the publisher.
