# Supplementary figures and images for: Distinct impacts of fat and fructose on the liver, muscle, and adipose tissue metabolome: An integrated view
Source: Front Endocrinol (Lausanne). 2022 Aug 17;13:898471. doi: 10.3389/fendo.2022.898471 (PMC9428722; doi:10.3389/fendo.2022.898471)

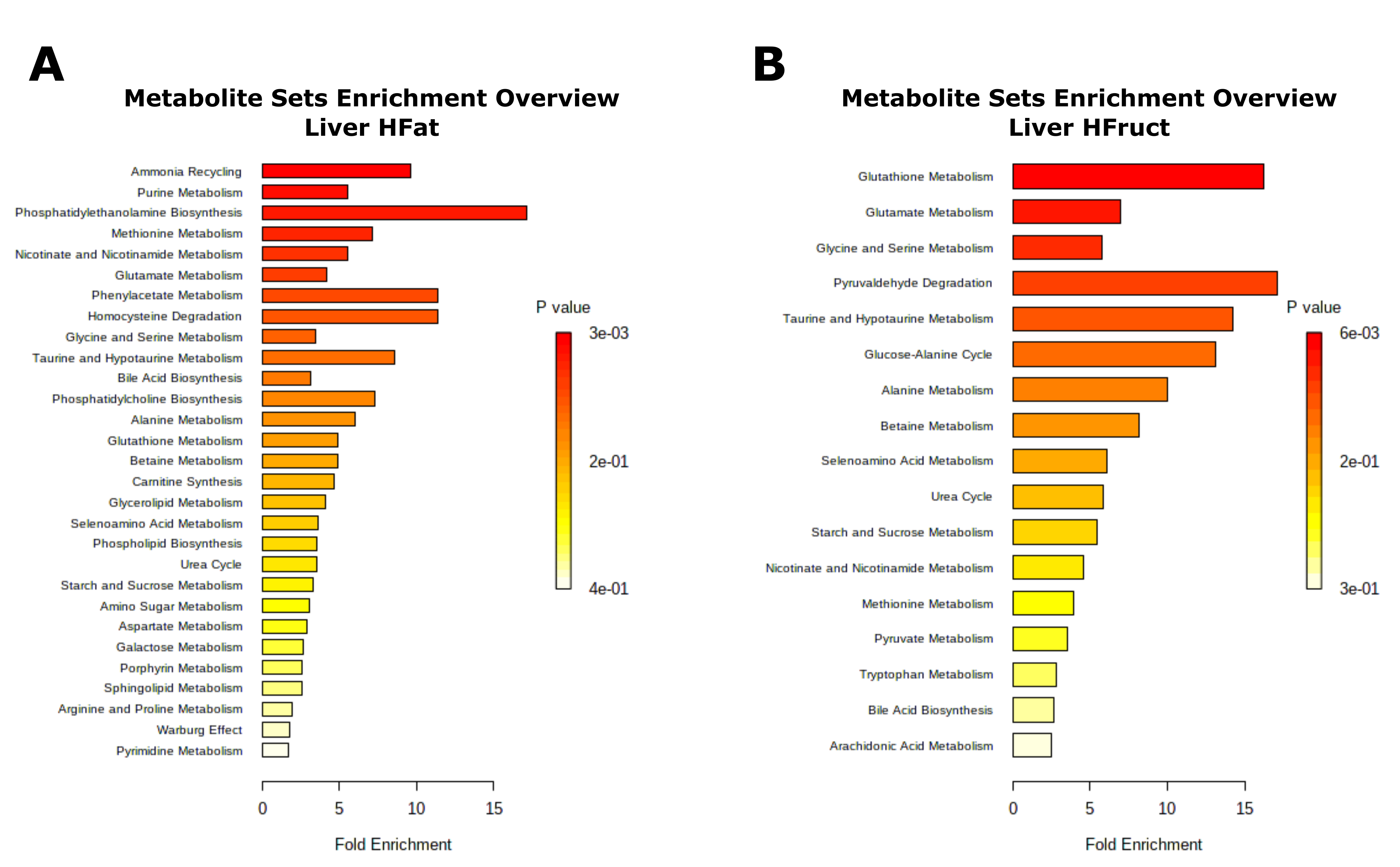

Supplement: Supplementary file 1 [file Image_1.tiff]

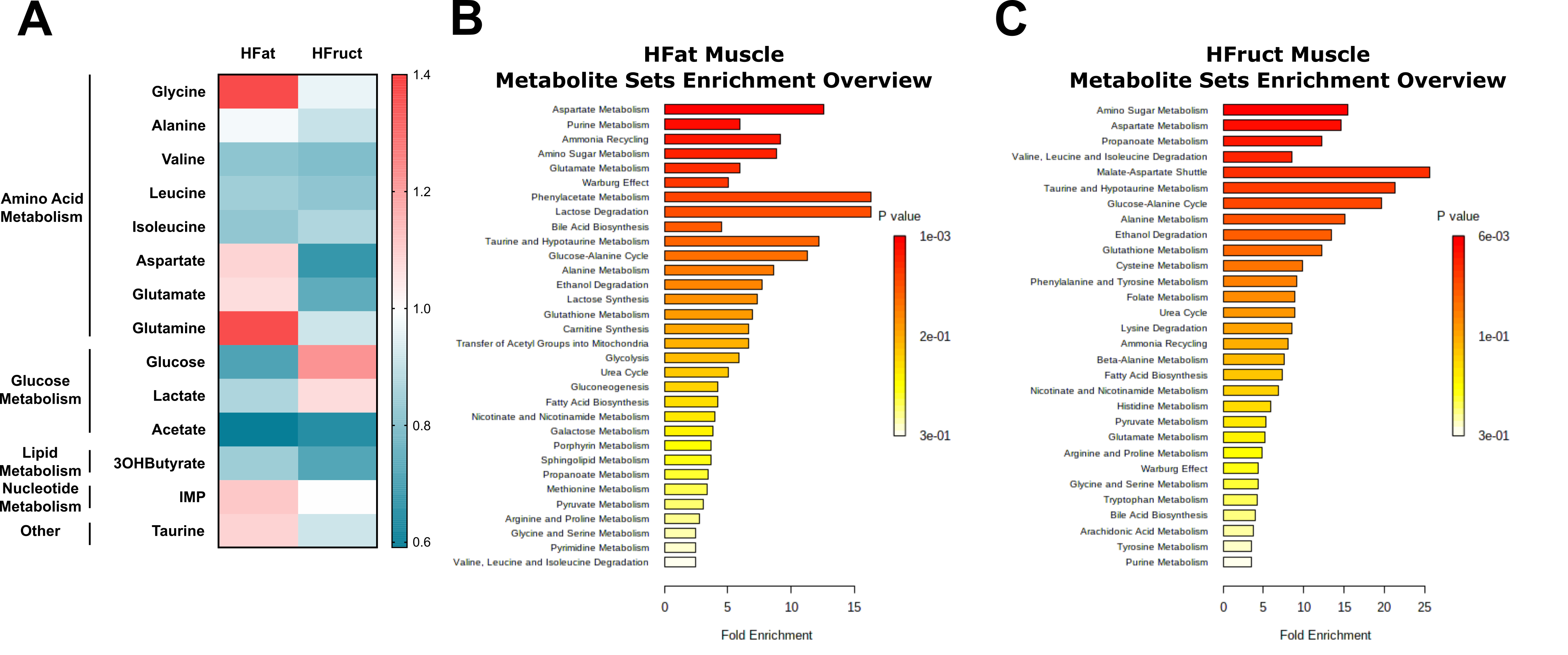

Supplement: Supplementary file 2 [file Image_2.tiff]

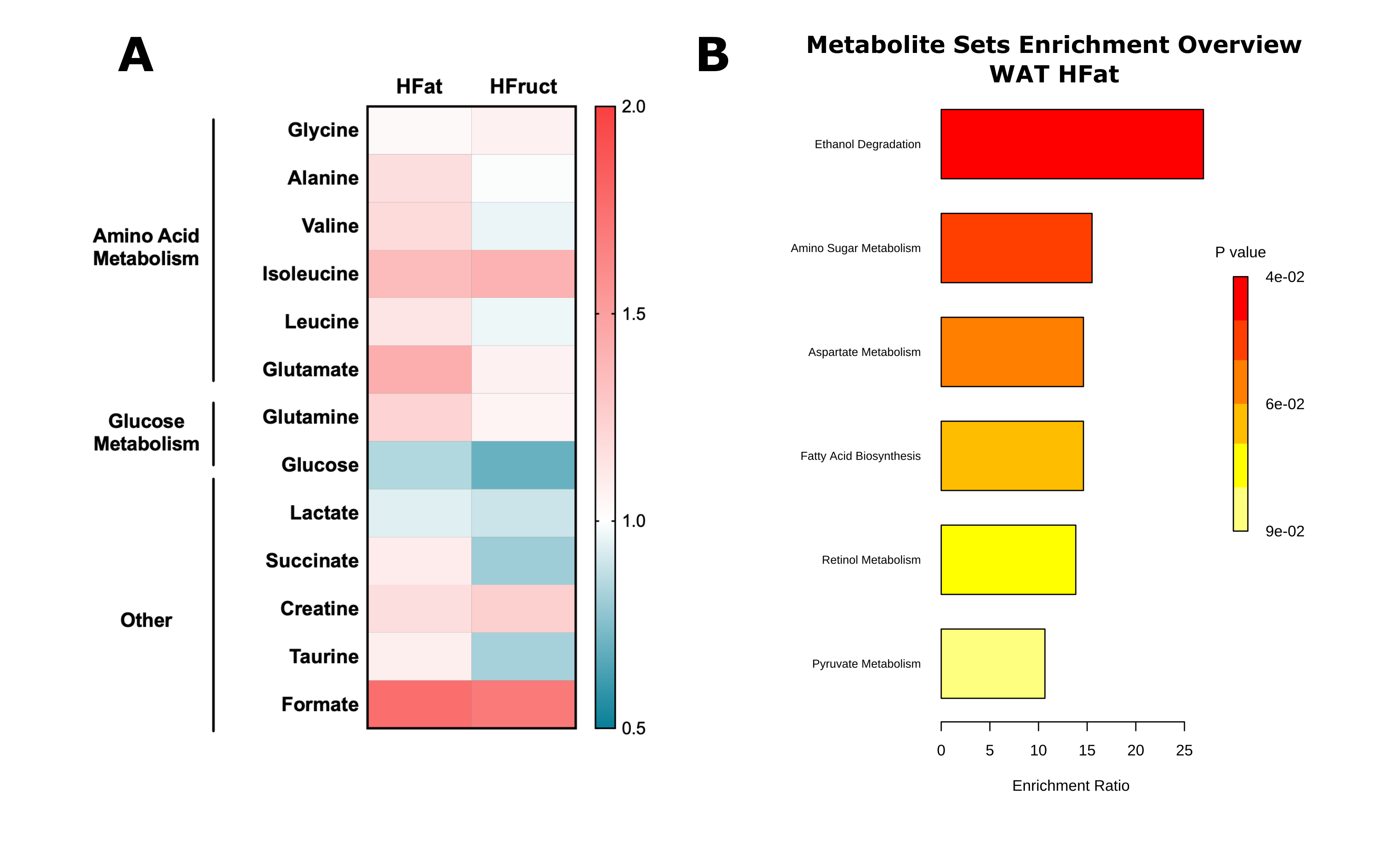

Supplement: Supplementary file 3 [file Image_3.tiff]

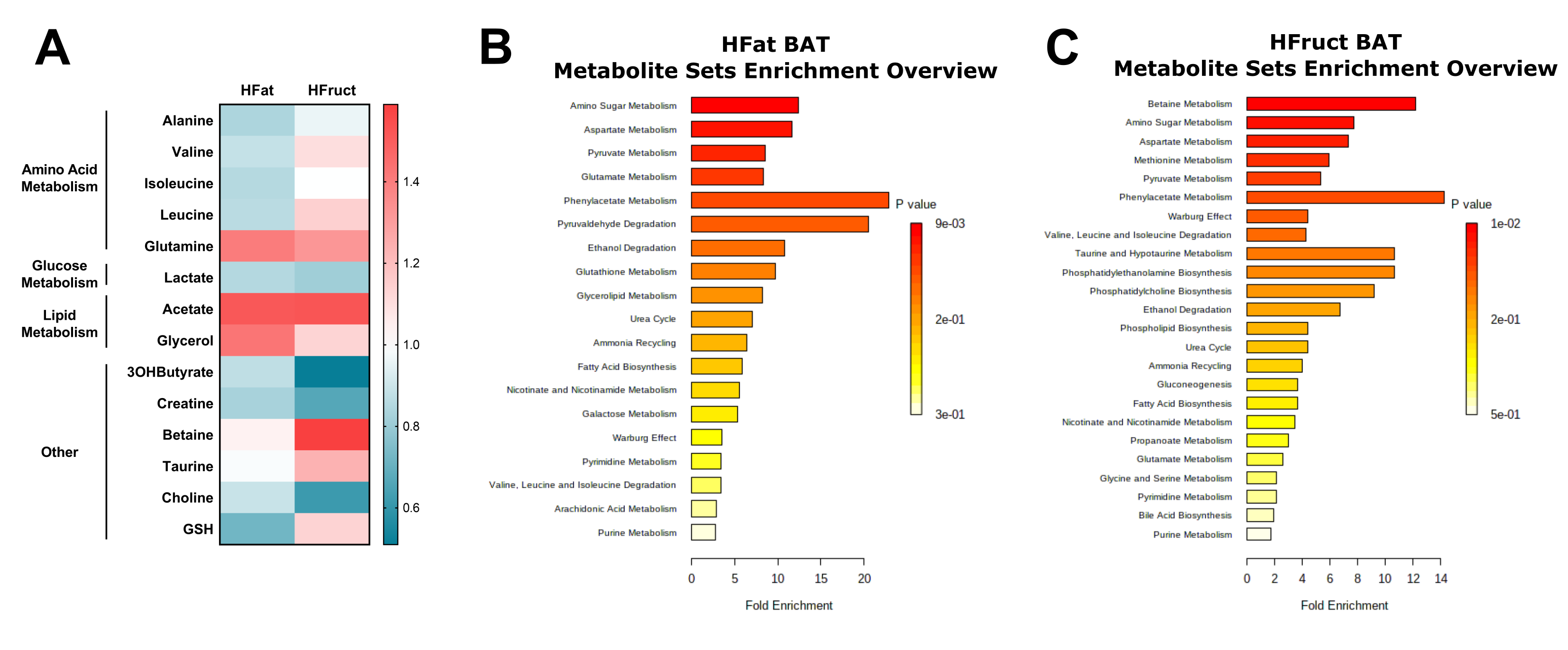

Supplement: Supplementary file 4 [file Image_4.png]

**LIVER**

**PCA**

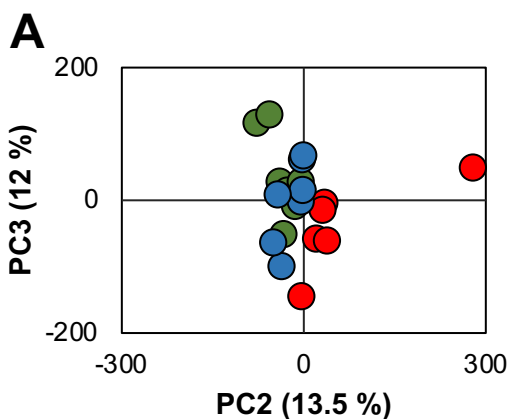

**PLS-DA**

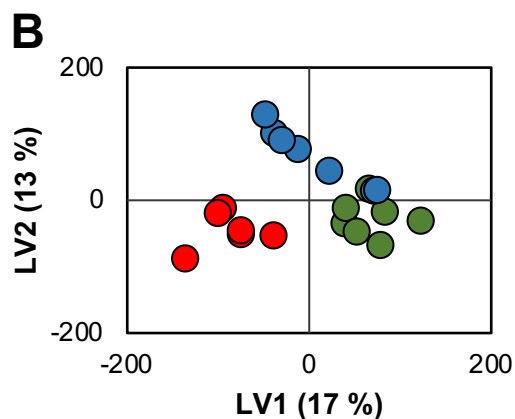

**MUSCLE**

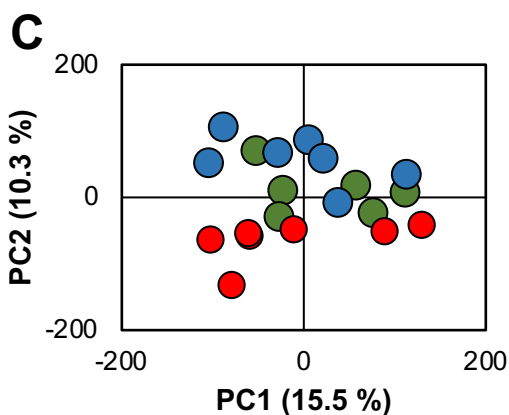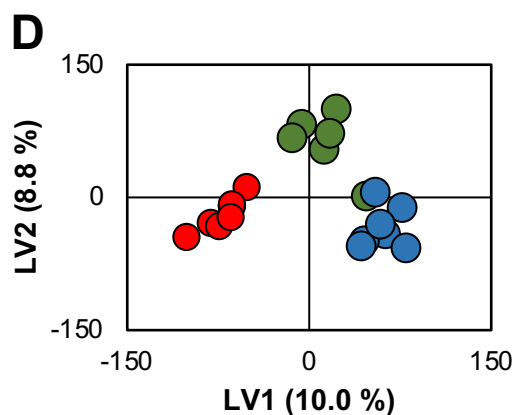

**WAT**

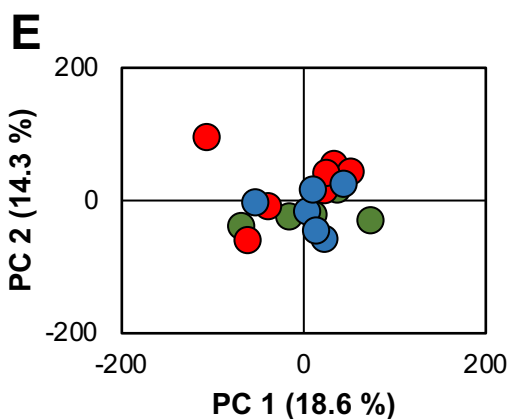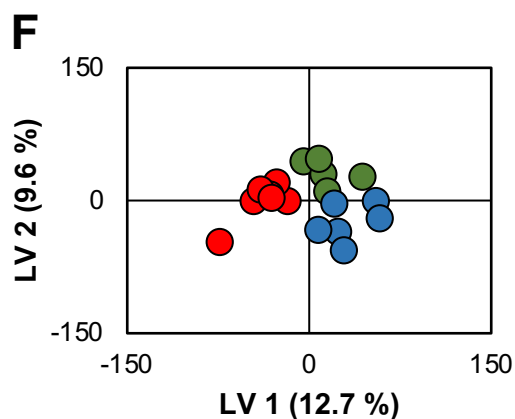

**BAT**

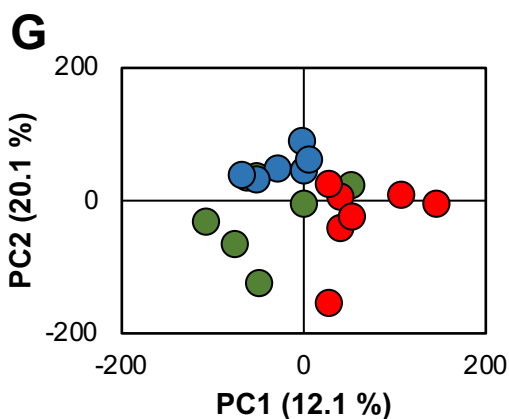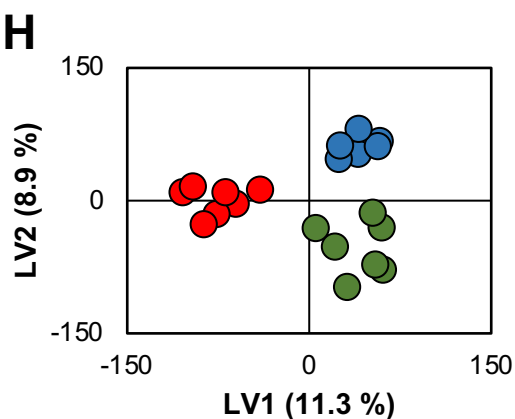

● Chow ● HFat ● HFruet

Supplement: Supplementary file 5 [file Image_5.pdf]
