# Supplementary material for: Distinct impacts of fat and fructose on the liver, muscle, and adipose tissue metabolome: An integrated view
Source: Front Endocrinol (Lausanne). 2022 Aug 17;13:898471. doi: 10.3389/fendo.2022.898471 (PMC9428722; doi:10.3389/fendo.2022.898471)
Supplement: Supplementary file 6 [file Table_1.docx]

# Supplementary Tables

# Supplementary Table 1 – List of antibodies used to evaluate the impact of high-fat and high-fructose diets on the insulin signaling pathway.

| Antibody | Host Specie | Molecular Weight | Dilution | Vendor | Catalog # |
| --- | --- | --- | --- | --- | --- |
| **InsR-α** | Rabbit | 95 kDa | 1:1000 | Cell Signaling | 74118 |
| **pInsR** | Rabbit | 95 kDa | 1:1000 | ThermoFisher Scientific | 44-806G |
| **Akt** | Rabbit | 60 kDa | 1:1000 | Santa Cruz Biotechnology | sc-7582 |
| **pAkt _S473_** | Rabbit | 60 kDa | 1:1000 | Cell Signaling Technology | 4060 |
| **pAkt _T308_** | Rabbit | 60 kDa | 1:1000 | Cell Signaling Technology | 4056 |
| **PON1** | Rabbit | 43 kDa | 1:2000 | Abcam | ab126597 |
| **GAPDH** | Mouse | 37 kDa | 1:10000 | Ambion | AM4300 |
| **β-Actin** | Mouse | 42 kDa | 1:5000 | Cell Signaling Technology | 3700 |
| **Mouse** | Goat | — | 1:5000 | Santa Cruz Biotechnology | sc-2031 |
| **Rabbit** | Goat | — | 1:5000 | Santa Cruz Biotechnology | sc-2030 |

# Supplementary Table 2 – Primer sequences and cycling conditions for polymerase chain reactions to evaluate the impact of high-fat and high-fructose diets in gene expression. Primer sequences of glucokinase (GK), glucose-6-phosphatase (G6Pase), phosphoenolpyruvate carboxykinase (PEPCK), carbohydrate-responsive element-binding protein (ChREBP), sterol regulatory element-binding protein 2 (SREBP2), elongation of very long chain fatty acids protein 2 (ELOVL2), cluster of differentiation 36 (CD36), stearoyl-CoA desaturase (SCD1), β-actin and β-2-microglobulin.

| Gene | Sequence 5’ -3’ | Annealing Temperature | Cycles |
| --- | --- | --- | --- |
| Glucokinase (NM_010292.5) | Fwd: GCTGGTACGACTTGTGCTG | 60°C | 35 |
|  | Rvs: TGGACACGCTTTCACAGG |  |  |
| G6Pase (NM_008061.4) | Fwd: ATGAACATTCTCCATGACTTTGGG | 58°C | 35 |
|  | Rvs: GACAGGGAACTGCTTTATTATAGG |  |  |
| PEPCK (NM_011044.3) | Fwd: TGAAAGGCCGCACCATGTAT | 60°C | 35 |
|  | Rvs: GCACAGATATGCCCATCCGA |  |  |
| ChREBP (NM_001359237.1) | Fwd: CTGGGGACCTAAACAGGAGC | 60°C | 35 |
|  | Rvs: GAAGCCACCCTATAGCTCCC |  |  |
| SREBP2 (NM_033218.1) | Fwd: GCGTTCTGGAGACCATGGA | 60°C | 35 |
|  | Rvs: CACAAGTTGCTCTGAAAACAAATCA |  |  |
| Elovl2 (NM_001311121.1) | Fwd: CCTGCTCTCGATATGGCTGG | 60°C | 35 |
|  | Rvs: AAGAAGTGTGATTGCGAGGTTAT |  |  |
| CD36 (NM_001159558.1) | Fwd: GATGACGTGGCAAAGAACAG | 58°C | 35 |
|  | Rvs: TCCTCGGGGTCCTGAGTTA |  |  |
| SCD1 (NM_009127.4) | Fwd: CGCCCCTACGACAAGAACAT | 60°C | 35 |
|  | Rvs: CTCAGAAGCCCAAAGCTCAG |  |  |
| β-actin (NM_007393.5) | Fwd: CGTGAAAAGATGACCCAGATCA | 60°C | 35 |
|  | Rvs: CACAGCCTGGATGGCTACGT |  |  |
| β-2-microglobulin (NM_009735.3) | Fwd: TCTCACTGACCGGCCTGTAT | 60°C | 35 |
|  | Rvs: CAGTCTCAGTGGGGGTGAAT |  |  |

**Supplementary Table 3 - Fatty acid composition of the diets used in this study.**

| Fatty Acid | Chow (%) | HFat (%) |
| --- | --- | --- |
| C6, Caproic | 0 | 0.20 |
| C8, Caprylic | 0 | 2.57 |
| C10, Capric | 0 | 1.97 |
| C12, Lauric | 0.05 | 15.87 |
| C14, Myristic | 0.20 | 6.00 |
| C14:1, Myristoleic | 0.01 | 0 |
| C16, Palmitic | 0.36 | 3.16 |
| C16:1, Palmitoleic | 0.13 | 0 |
| C18, Stearic | 0.09 | 3.63 |
| C18:1, Oleic | 1.03 | 0.87 |
| C18:2, Linoleic | 1.15 | 1.35 |
| C18:3, Linolenic | 0.17 | 0.20 |
| C20:4, Arachidonic | 0.22 | 0 |
| C22:5, Docosapentaenoic | 0.04 | 0 |

# Supplementary Data Figures

**Supplementary Figure 1** – Effects of the HFat and HFruct diets on the hepatic metabolome of C57Bl/6J male mice. Hepatic metabolite sets enrichment overview of HFat (Panel A) and HFruct mice (Panel B).

**Supplementary Figure 2** – Effects of the HFat and HFruct diets on the muscle metabolome of C57Bl/6J male mice. Heat map of muscle polar metabolites in comparison to Chow metabolites, which are represented by 1/white (Panel A), and muscle metabolite sets enrichment overview of HFat (Panel B) and HFruct mice (Panel C).

**Supplementary Figure 3** – Effects of the HFat and HFruct diets on the white adipose tissue (WAT) metabolome of C57Bl/6J male mice. Heat map of WAT polar metabolites in comparison to Chow metabolites, which are represented by 1/white (Panel A), and WAT metabolite sets enrichment overview of HFat (Panel B).

**Supplementary Figure 4** – Effects of the HFat and HFruct diets on the brown adipose tissue (BAT) metabolome of C57Bl/6J male mice. Heat map of BAT polar metabolites in comparison to Chow metabolites, which are represented by 1/white (Panel A), and BAT metabolite sets enrichment overview of HFat (Panel B) and HFruct mice (Panel C).

**Supplementary Figure 5** – Multivariate analysis of liver, muscle, white and brown adipose tissue polar metabolites. A – Liver principal component analysis (PCA) scatter scores plot of Chow, high-fat (HFat) and high-fructose (HFruct) groups; B – Liver partial least square-determinant analysis (PLS-DA) scatter scores plot of Chow, HFat and HFruct groups (permutation test; n=100; R^2^=0.94, Q^2^=-0.15); C – Muscle PCA scatter scores plot of Chow, HFat and HFruct groups; D – Muscle PLS-DA scatter scores plot of Chow, HFat and HFruct diets (permutation test; n=100; R^2^=0.76, Q^2^=-0.066); E – White adipose tissue (WAT) PCA scatter scores plot of Chow, HFat and HFruct diets; F – WAT PLS-DA scatter scores plot of Chow, HFat and HFruct diets (permutation test; n=100; R^2^=0.982, Q^2^=0.115); G – Brown adipose tissue (BAT) PCA scatter scores plot of Chow, HFat and HFruct diets; H – BAT PLS-DA scatter scores plot of Chow, HFat and HFruct diets (permutation test; n=100; R^2^=0.982, Q^2^=0.115).
